# Adherence to American Heart Association and American College of Cardiology guidelines for exercise tolerance test in cardiovascular clinics

**DOI:** 10.15171/jcvtr.2019.49

**Published:** 2019-10-24

**Authors:** Nahid Hatam, Vida Razazi Khales, Mehrdad Askarian, Abdolali Zolghadrasli, Shohre Hooshmand, Mohammadali Ostovan

**Affiliations:** ^1^Department of Health Service Administration, School of Management and Information Sciences, Shiraz University of Medical Sciences, Shiraz, Iran; ^2^Student Research Committee, Department of Health Service Administration, School of Management and Information Sciences, Shiraz University of Medical Sciences, Shiraz, Iran; ^3^Department of Community Medicine, School of Medicine, Shiraz Nephro-Urology Research Center, Shiraz University of Medical Sciences, Shiraz, Iran; ^4^Department of Cardiology, School of Medicine, Shiraz University of Medical Sciences, Shiraz, Iran; ^5^Shiraz Cardiovascular Research Center, Shiraz, Iran; ^6^Department of Cardiology, Shiraz University of Medical Sciences, Shiraz, Iran

**Keywords:** Appropriate Use Criteria, Guideline, Exercise Test

## Abstract

***Introduction:*** Considering the increased expenditure in public health sector, especially the increased cost in hospitals and clinics, there is an urgent need to control these costs mainly by ensuring adherence to clinical guidelines for diagnostic procedures. In this study we aim to investigate the adherence of heart clinics to guideline for exercise tolerance test.

*** Methods:*** This cross-sectional study was performed on 308 patients who were referred for ECG exercise test in 3 clinics located in the city of Shiraz, Iran in 2018. Demographic and clinical data were recorded and the indications of exercise test for each patient was reviewed according to the ACC/AHA guideline for exercise tolerance test.

***Results:*** Exercise tests were found to be inappropriately done in 121 (39.3%) participants. Among the patients for whom the test was done without indication 79 (65.3%) were women and the gender difference was statistically significant (*P* < 0.01); women were 18.5% more likely to undergo exercise test without indication. There was more inappropriate tests among nonanginal pain subsets comparing to other presenting symptoms (*P* < 0.001). Age, coronary risk factors, reason for performing exercise tests and private health system were not predictors of inappropriate use (*P* > 0.05).

*** Conclusion:*** This study confirms that more than one third of exercise tests done in the participants are inappropriate. Wide availability of exercise test makes it vulnerable to overuse and additional unnecessary cost to health care systems.

## Introduction

According to World Health Organization reports, the costs of public health in hospitals and clinics has increased in most countries and is a burden on the public sector, which threatens the overall integrity of the system.^1^ Management of the current resources without increasing the expenses is a great challenge which affects patients and health managers equally.^2^ This is of special concern in developing countries where already strained resources are increasingly being directed toward health sector.^3,4^ There are several solutions to this increased expenditure, including constant surveillance, establishment of a robust referral system and adherence to clinical guidelines established at international, national and institutional levels.^4-6^ As mentioned earlier, ensuring adherence to clinical guidelines is one method of controlling the spiraling health expenditure. This can be particularly true in the management of chronic diseases which are considered one of the most draining subjects of health budgets.^7^ Among chronic diseases, coronary artery disease is the leading cause of death worldwide, and annual direct and indirect cost of cardiovascular disease in the United states of America is estimated to be around $350 billion,^8^ and Iran ranks 25 in the mortality rate of coronary artery diseases.^9^ Exercise tolerance test is one of the most common and basic tools used by cardiologists to evaluate the possibility and severity of coronary artery disease.^10^ Worldwide data shows that inappropriate exercise tolerance test was prescribed for more than half of the cases.^11-13^ Considering the widespread use of this modality and the inherent risks associated with its performance and interpretation, we decided to study the adherence of heart clinics in Shiraz, Iran to American Heart Association and American College of Cardiology guideline for exercise test.

## Materials and Methods

This cross-sectional descriptive study was performed in 2018 in the city of Shiraz, Iran. Three clinics were selected for this study with the simple random sampling method. One of them was private and the other two were public clinics. Initially 17 patients were selected from each clinic and a pilot study was done. Considering d = 0.55 and *P* = 0.39 and error = 5%, the final sample size was calculated at 303 people.

The exercise test guideline published in 2002 by the American Heart Association and American College of Cardiology was used to determine the standard against which the indications and contraindications of exercise test were determined.^14^ The guideline was translated from English into Persian and then back into English and checked for reliability by 3 cardiologists. Then a checklist was extracted from the guideline to determine the indications and contraindications of exercise test. Thereafter a questionnaire was used which was composed of 3 parts: (1) demographic data of the patient, (2) past medical history of the patient, presenting symptoms, ECG and results of previous studies, (3) the indication for and the results of the exercise test. All patients gave informed consent and care was given not to hamper with the normal function of the heart clinic.

The patients who were referred for exercise test were interviewed and the questionnaires were filled for them. Their ECGs and the results of their exercise test were recorded and two cardiologists reviewed the questionnaire and determined the adherence to the guidelines according to the checklist provided, considering factors such as baseline ECG, type of chest pain, history of revascularization, indications and contraindications to exercise test.

In this study descriptive analytical data like percentage was used to present the data. Chi-square test was used to analyze qualitative data and Fisher’s exact test was used to analyze quantitative data. *P* value less than 0.05 was considered significant. SPSS version 18 was used in this study.

## Results

The mean age of the participants was 52.7 years. There were 165 women (53.6%) and 143 men (46.4%). Moreover, 65.2% of tests were done in public healthcare system. Other demographic and clinical history of study participants are depicted in Table 1. Among the participants, 37 (12%) had typical chest pain, 126 (41%) had atypical chest pain, 16 (5%) had non-anginal pain and 129 (42%) had no chest pain at all.106 patients (34.4%) suffered from palpitation, 15 (4.9%) suffered from syncope or presyncope, 91 (29.5%) complained of dyspnea and 179 (58.1%) had chest pain as their main presenting symptoms. Table 2 shows the indications for performing exercise test and other test characteristics in the study subjects. Among the participants 187 people (60.7%) had discontinued their medications before the test, while 121 (39.3%) did not discontinue their medications. According to our final assessment, out of the 308 participants, 187 cases underwent an exercise test according to the guidelines (60.7%) and 121 participants (39.3%) underwent exercise test without guideline indications. Among the tests done without indication, 79 (65.3%) were women and 42 (34.7%) were men and the gender difference was statistically significant (*P*< 0.01); women were 18.5% more likely to undergo an exercise test without indication. The mean age of patients who underwent test without indications was 53.2 years and those without indications was 50.2 years, which was not statistically significant (*P* = 0.11). The results were also interpreted according to the presenting symptoms which showed that those with non-anginal chest pain are more likely to undergo an exercise test without indication (12.5 % with indications vs 87.5% without indications) (*P* < 0.001). Of note, exercise tests obtained from public clinics were not significantly more appropriate than private sector clinics (*P* = 0.21). On the other hand, there was no relation between adherence to guideline and the presenting symptoms of the patients (chest pain, palpitation, syncope or dyspnea) (*P* = 0.16), nor with the reason for performing the exercise test (*P* = 0.12) or coronary disease risk factors (*P* >0.05) as shown in Tables 3 and 4.

**Table 1:** Personal characteristics of study subjects

**Parameters**	**No. (%)**
N	308
Age (y)	52.7 ± 12.2
Male	143 (46.4)
Smoker	103 (33.4)
Hypertension	122 (39.6)
Diabetes	131 (42.5)
Dyslipidemia	125 (40.5)
Obesity	77 (25)
Family history of CAD	114 (37)
Known CAD	151 (49)
Revascularization	144 (46.7)
**Types of chest pain**	
No chest pain	129 (42)
Typical angina	37 (12)
Atypical angina	126 (41)
Nonanginal	16 (5)
Test of public health system	201 (65.2)
Test of private health system	107 (34.8)

**Table 2:** Exercise tests characteristics of study subjects

**Parameters**	**No. (%)**
Indication for ETT	
Diagnosis of CAD	98 (31.8)
Preoperative evaluation	19 (6.1)
Ischemia after revascularization	85 (27.5)
Planning after catheterization	45 (14.6)
Prognosis & outcome	61 (19.8)
Test results	
Negative	208 (67.5)
Low risk positive	45 (14.6)
Highly positive	14 (4.5)
Ventricular tachycardia	1 (0.3)
Equivocal	40 (12.9)
Baseline ECG	
Normal	216 (70.1)
LBBB	1 (0.3)
RBBB	11 (3.5)
LVH	10 (3.2)
ST depression> 1 mm	**64 (20.7)**

**Table 3:** Patients characteristics according to exercise test appropriateness criteria

**Parameter**	**Appropriate** **(n= 187)**	**In appropriate** **(n=121)**	***P*** **value**
Male	101 (54)	42 (34.7)	0.01
Age	53.2 ± 12	50.2 ± 13	0.11
Smoker	60 (32)	43 (35.5)	0.63
Hypertension	75 (40.1)	47 (38.8)	0.67
Diabetes	80 (42.7)	51 (42.1)	0.71
Dyslipidemia	75 (40.1)	50 (41.3)	0.68
Obesity	46 (24.5)	31 (25.6)	0.65
Family history of CAD	64 (34.2)	50 (41.3)	0.25
Known CAD	93 (49.7)	58 (47.9)	0.73
Revascularization	89 (47.5)	55 (45.4)	0.52
**Types of chest pain**			<0.001
No chest pain	77 (59.7)	52 (40.3)	
Atypical angina	75 (59.5)	51 (40.5)	
Typical angina	22 (59.4)	15 (40.6)	
Nonanginal	2 (12.5)	14 (87.5)	
Test of public health system	128 (68.5)	73 (60.3)	0.21

**Table 4:** Exercise test indications and adherence to ACC/AHA guidelines

**Indication**	**Adherence** **(n=187)**	**Nonadherence** **(n=121)**	***P*** **value**
Diagnosis of CAD	64 (34.2)	34 (28)	0.19
Preoperative evaluation	11 (5.8)	8 (6.6)	
Ischemia after revascularization	49 (26.2)	36 (24.7)	
Planning after coronary angiography	28 (14.9)	17 (14)	
Prognosis & outcome	35 (17.8)	26 (21.4)	

## Discussion

There is increasing evidence that in recent years there has been an increase in the number of expensive and sometimes unnecessary diagnostic tests, so studies to assess the adherence to guidelines is definitely necessary. In Iran only a few such studies have been done, for example, in one study published in 2013 on 280 patients, it was determined that only 60% of all coronary angiographies were performed according to the American Heart Association and American College of Cardiology guidelines.^15^ Our study is the first to investigate the adherence to exercise test as one of the most common diagnostic modalities performed by cardiologists in Iran. In our study the mean age of the patients was 52.7 years. The most common coronary risk factor was diabetes mellitus (42.5%) and the least common was smoking (33.4%). The most common indication for exercise test was diagnosis of coronary artery disease and the exercise test was deemed to be not according to the guidelines in 39.3% of cases and the inappropriate cases were more likely to be women and those with non-anginal chest pain. A total of 5% of our patients had non-anginal chest pain and 87.5% of them had inappropriate exercise test, so it is important to consider them carefully before sending them to exercise test. The same result was concluded by Gertz et al who demonstrated that there is a positive relationship between the type of chest pain and the appropriateness of exercise test.^16^ Gertz et al also demonstrated that there is no relationship between appropriateness of exercise test and male gender, but the study done by Sagarad et al demonstrated that women are more likely to be sent to exercise test inappropriately,^13^ this result was in line with our conclusion. Sagarad et al demonstrated a 49.66% inappropriate exercise test in their study in 2016 which was attributed to availability and low cost of exercise test.^13^ Gertz et al found in their study that 13% of exercise tests were inappropriate according to guidelines.^16^ The much lower rate in their study was most likely because of the setting in which it was conducted which was a hospital setting. Bilal et al^12^ in their study in 2018 concluded that 79% of exercise tests were not done according to guidelines and they attributed this finding to the practices of private sector clinics. In our study, age, coronary risk factors, reason for performing exercise tests and private health system were not predictors of inappropriate use of the exercise test. Apparently the very low cost of exercise tests in our public health systems compensate its overusage in private health sector clinics.

## Conclusion

Exercise tolerance test iswidely available and is more prone to being used beyond the recommended guidelines because of the lack of enough awareness and/or any other reasons. The results of this study showed a high prevalence of inappropriate exercise test in diagnosis and management of coronary artery disease. There is a serious need to promote awareness among cardiologists and to follow the recommended guidelines and form an understanding that favor the careful use of medical resources even when they are largely available, focusing on the guidelines and the appropriate use of the complementary diagnostic methods.

## Competing interests

None.

## Ethical approval

Informed consent was taken from patients.

## Acknowledgements

This research was performed by Ms. Vida Razazi Khales in partial fulfillment of the requirements for certification as an MSc in Health Care Management, School of Management and Information Sciences, Shiraz University of Medical Sciences, Shiraz, Iran. The present article was adopted from the proposal number: 95-13289 approved by vice-chancellor for research affairs of Shiraz University of Medical Sciences.
